# Design SMAP29-LysPA26 as a Highly Efficient Artilysin against Pseudomonas aeruginosa with Bactericidal and Antibiofilm Activity

**DOI:** 10.1128/Spectrum.00546-21

**Published:** 2021-12-08

**Authors:** Tingting Wang, Yongxiang Zheng, Jiami Dai, Junxiu Zhou, Rong Yu, Chun Zhang

**Affiliations:** a Department of Biopharmaceutics, West China School of Pharmacy, Sichuan University, Chengdu, China; b Key Laboratory of Drug-Targeting and Drug Delivery System of the Education Ministry, Sichuan Engineering Laboratory for Plant-Sourced Drug and Sichuan Research Center for Drug Precision Industrial Technology, West China School of Pharmacy Sichuan University, Chengdu, China; University of Pittsburgh School of Medicine

**Keywords:** endolysin, LysPA26, SMAP29, AL-3AA, *Pseudomonas aeruginosa*, antimicrobial agent, antimicrobial resistance, Gram-negative infection, bactericidal, biofilm, circular dichroism, protein design

## Abstract

Antimicrobial resistance (AMR) is a major issue to global health. The multidrug-resistant (MDR) Gram-negative infections, particularly infected by carbapenem-resistant pathogens, urgently need efficient antibiotics and novel therapy. However, the scientific challenges of aiming for innovative approaches against Gram-negative bacteria have hindered the research and development of antibiotic drugs. Phage-derived endolysins are bacteriolytic and specific for a bacterial species or genus, providing a promising antibiotic strategy. However, the outer membrane of Gram-negative bacteria could prevent the peptidoglycan layer from the hydrolysis of endolysins. Antimicrobial peptides usually destabilize the outer membrane and could enhance the antibiotic activity of endolysins. In this study, we designed new artilysins with antimicrobial-peptide SMAP29 fusion at the N-terminal of LysPA26 (named as AL-3AA, AL-9AA, and AL-15AA), and evaluated them. The results showed artilysin AL-3AA to be highly bactericidal; even 0.05 mg/mL AL-3AA could reduce 5.81 log units P. aeruginosa without EDTA in 60 min. It killed P. aeruginosa rapidly and dose-dependently through cell lysis. AL-3AA inhibited P. aeruginosa PAO1 biofilm formation and significantly decreased mature P. aeruginosa biofilms. It also had potential broad-spectrum activity against susceptible Gram-negative bacteria in the hospital, including K. pneumoniae and E. coli. The antibacterial mechanism investigation has provided valuable information about the antibacterial action of AL-3AA, which can lyse and disintegrate the bacterial quickly. These results suggested AL-3AA could be a new and promising antimicrobial agent for the combat of P. aeruginosa.

**IMPORTANCE** Antimicrobial resistance (AMR) is a major issue to global health, particularly the multidrug-resistant (MDR) Gram-negative infections, which pose great challenges. Even new antibiotics research is ongoing, antibiotics used to treat Gram-negative bacteria in the clinical are limited in a small set of molecular scaffolds, and biomolecular categories of antibiotics are urgently needed. In this study, we designed new proteins by combining antimicrobial peptides and endolysins for synergistic bactericidal effects. One of designed proteins, named AL-3AA, showed highly bactericidal, and killed P. aeruginosa rapidly and dose-dependently through cell lysis. It also killed Klebsiella pneumoniae and Escherichia coli, showing potential broad-spectrum activity against susceptible Gram-negative bacteria in the hospital. All results suggest AL-3AA could be a new and promising antimicrobial agent for the combat of P. aeruginosa.

## INTRODUCTION

Antimicrobial resistance (AMR), induced by antibiotic use and overuse, is a major issue to global health (1–3). Especially, the multidrug-resistant (MDR) Gram-negative infections, particularly infected by carbapenem-resistant pathogens such as Pseudomonas aeruginosa and Klebsiella pneumoniae, urgently need efficient antibiotics and novel therapies (4, 5). However, the antibiotics used to clinically treat Gram-negative bacteria are limited in a small set of molecular scaffolds, reflecting the scientific challenges of aiming for innovative approaches and the lower research risk (6, 7). Novel antibiotic agents against resistant P. aeruginosa, A. baumannii and *Enterobacteriacea*, which are the critical priority pathogens based on the WHO Priority Pathogen List, are thus desperately needed (6).

Most clinically-used antibiotics inhibit enzymes from pathways such as peptidoglycan synthesis, ribosomal protein synthesis, folate synthesis, and nucleic acid synthesis and topoisomerization (8). They inhibit or kill the bacteria, however, on the other hand, they promote developing resistance to antibiotics. Biomolecular categories of antibiotic strategies have received increasing attention in recent years, including phage therapy and phage-derived proteins (7, 9). Phage therapies are species specific, and the most researched programs were therapies targeting P. aeruginosa and S. aureus (10, 11). However, compared with small molecule antibiotics, the enormous size of phages imposes pharmacokinetic challenges and a risk of phage-neutralizing antibodies (12).

In contrast to the great size of phages, phage-derived proteins such as endolysins have attracted increasing research attention (13–16). Endolysins are bacteriolytic and specific for bacterial species, through cleaving the peptidoglycan layer of the cell wall. The promising antibacterial effect of endolysins has been intensively studied and confirmed *in vitro* and in animal models of infection (9, 15, 17). In the life cycle of phages, endolysins naturally attack the cell wall from the inside out with the help of holin, thus the activity of extracellular added endolysins as treatments are usually blocked by the outer membrane of Gram-negative bacteria (18, 19). Even some endolysins, such as LysPA26 and KZ144 (16, 20), were reported with the inhibition against Gram-negative bacteria; their antibiotic effect was enhanced by membrane destabilizing agents, such as EDTA (16, 20), indicating improving the entry of endolysins into the peptidoglycan layer can increase the antibacterial effect of endolysins. It is well known that antimicrobial peptides usually kill bacteria through membrane permeability and insertion, even pore formation (21–24). Thus, antimicrobial peptides were fused on the terminal of endolysins to increase the access to the peptidoglycan layer. One example is Art-175, with a sheep myeloid 29 amino acid peptide (SMAP-29) fused on the N-terminal of KZ-144 (25). Although, both KZ-144 and LysPA26 could kill P. aeruginosa, LysPA26 has a broader antimicrobial spectrum, such as P. aeruginosa, Klebsiella pneumonia, Acinetobacter baumannii, and Escherichia coli (16, 20). The antimicrobial peptides and LysPA26 fusion protein may enhance the antibacterial activity of parental enzybiotics, through targeting to both the membrane and peptidoglycan layer. Linkers are usually used to design one fusion protein with two functional domains or proteins. The peptide rigidity and length of linkers have impact on activity of fusion proteins, and flexible linkers are usually applied when the joined domains require a certain degree of movement or interaction. Thus, flexible linkers, such as (GSA)_n_ and (GGGGS)_n_, were used to link SMAP29 and LysPA26 in this study, aiming to realize synergistic effects of SMAP29 and LysPA26.

To identify new dual-target antibiotic proteins, we have designed three Antimicrobial-peptides-Lysin (AL) fusion proteins, with different linker, named SMAP29-GSA-LysPA26 (AL-3AA), SMAP29-(GSA)_3_-LysPA26 (AL-9AA), and SMAP29-(GGGGS)_3_-LysPA26 (AL-15AA). We have evaluated their antibiotic and antibiofilm effect, and investigated their active mode and mechanism. Our results showed AL-3AA has high antimicrobial activity against P. aeruginosa, kills P. aeruginosa rapidly through cell lysis, and prevents biofilm formation. It also has a broad-spectrum antimicrobial activity.

## RESULTS AND DISCUSSION

### SMAP29-LysPA26 (AL-3AA) efficiently kills P. aeruginosa.

**FIG 1:**
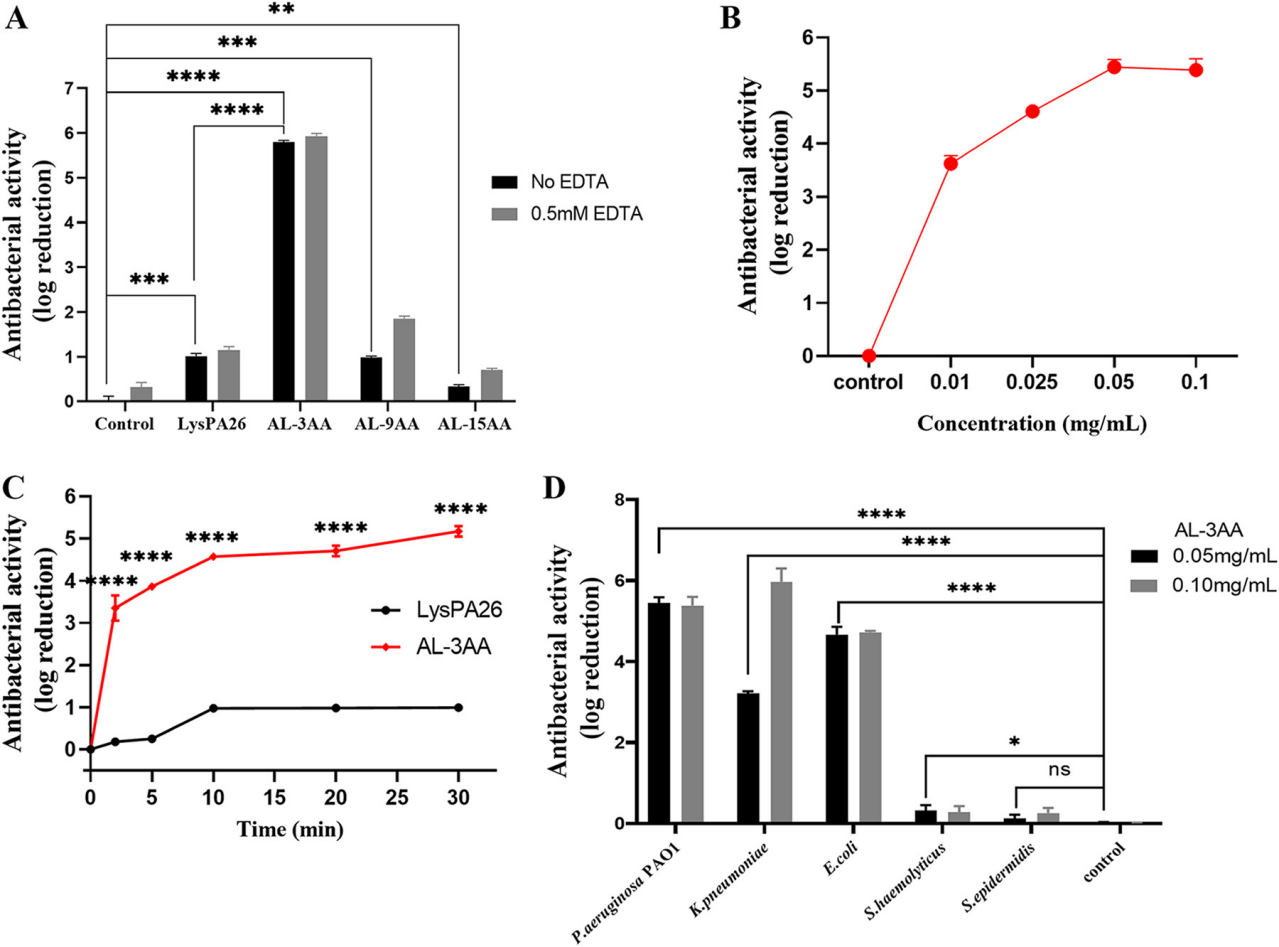
Bactericidal activity of AL-3AA, AL-9AA, AL-15AA, and LysPA26 against P. aeruginosa PAO1. (A) The antibacterial activity is expressed in log_10_ units, and mean values (standard deviation [SD]) are shown. (B) The dose-dependent antibacterial activity of AL-3AA. (C) The rapid killing activity of AL-3AA. (D) The antibacterial activity of AL-3AA (0.05 mg/mL and 0.10 mg/mL) against P. aeruginosa PAO1, K. pneumoniae, E. coli, S. haemolyticus, and S. epidermidis. Significant difference between groups is indicated by *, **, ***, and ****, representing *P* < 0.05, *P* < 0.01, *P* < 0.001, and *P* < 0.0001, respectively. (N = 3).

To improve the antibacterial activity of LysPA26, SMAP29 peptide was fused on the N-terminal of LysPA26, with different linkers, termed as SMAP29-GSA-LysPA26 (AL-3AA), SMAP29-(GSA)_3_-LysPA26 (AL-9AA), and SMAP29-(GGGGS)_3_-LysPA26 (AL-15AA). All the recombinant artilysins were expressed and purified. The antibacterial activity of AL-3AA, AL-9AA, AL-15AA, and LysPA26 at a concentration of 0.05 mg/mL against P. aeruginosa PAO1 was analyzed without or with 0.5 mM EDTA. As shown in Fig. 1A, LysPA26 showed only limited antibacterial activity, approximately 1.01 and 1.14 log units without or with 0.5 mM EDTA, respectively, similar to the reported data, probably because it cannot efficiently permeate the outer membrane to hydrolyze the peptidoglycan layer. In contrast to LysPA26, AL-3AA (0.05 mg/mL) killed more than 99.9995% P. aeruginosa PAO1 and showed notable bactericidal, 5.81 log units without EDTA in 60 min (Fig. 1A), which is 5.75-fold that of LysPA26 (Fig. S1). The AL-9AA had similar antibacterial activity to LysPA26, 0.98 log units without EDTA. However, the antibacterial activity of AL-15AA was notably reduced to 0.33 log units without EDTA (Fig. 1A). Comparing the activity of four proteins, the introduction of SMAP29 could enhance the bactericidal activity of LysPA26 significantly, and the linker impacts the bactericidal activity of artilysin. This impact was further analyzed through protein conformation changes.

### AL-3AA rapidly kills P. aeruginosa dose-dependently.

To illustrate the active mode of AL-3AA, P. aeruginosa PAO1 was incubated with gradient concentrations of AL-3AA for 1 h, and in another assay P. aeruginosa PAO1 was incubated with 0.05 mg/mL AL-3AA for different times. As shown in Fig. 1B, AL-3AA killed P. aeruginosa PAO1 dose-dependently. AL-3AA at 0.05 mg/mL can kill more than 99.999% P. aeruginosa PAO1 in 1 h, showing antibacterial activity approximately 5.44 log units; even 0.01 mg/mL AL-3AA killed more than 99.97% P. aeruginosa PAO1 in 1 h, showing antibacterial activity approximately 3.62 log units (Fig. 1B). At incubation for only 2 min, AL-3AA showed antibacterial activity approximately 3.36 log units (Fig. 1C), about 18-fold that of LysPA26 with the same incubation time (Fig. S3). When incubated for 30 min, it showed antibacterial activity approximately 5.17 log units (Fig. 1C). All the activity of AL-3AA at different incubation times were at least 4.71-fold higher than the corresponding group of LysPA26 (Fig. S3). As reported, Art-175 0.10 mg/mL showed antibacterial activity approximately 4.5 log units after a 30 min incubation (25), in this study, the 0.05 mg/mL AL-3AA showed antibacterial activity approximately 5.17 log units. Thus, AL-3AA could kill P. aeruginosa PAO1 at a low dose rapidly, showing good potential of clinic application in future.

### AL-3AA has potential broad-spectrum antimicrobial activity.

To determine the antibiotic spectrum, the inhibitory effect of AL-3AA against some hospital susceptible bacteria including Gram-negative and Gram-positive bacteria were investigated *in vitro*. AL-3AA showed antibacterial activity not only against P. aeruginosa PAO1, but also against Klebsiella pneumoniae and Escherichia coli (Gram-negative) (Fig. 1D). AL-3AA (0.10 mg/mL) showed antibacterial activity approximately 5.98 log units against K. pneumoniae and 4.72 log units against E. coli, respectively. However, AL-3AA did not obviously exhibit antibacterial activity against S. haemolyticus and S. epidermidis, both of which are Gram-positive bacteria. These data indicated AL-3AA has potential broad-spectrum activity against susceptible Gram-negative bacteria in the hospital.

### AL-3AA pretreatment reduces biofilm formation and significantly decreases mature P. aeruginosa biofilms *in vitro*.

**FIG 2:**
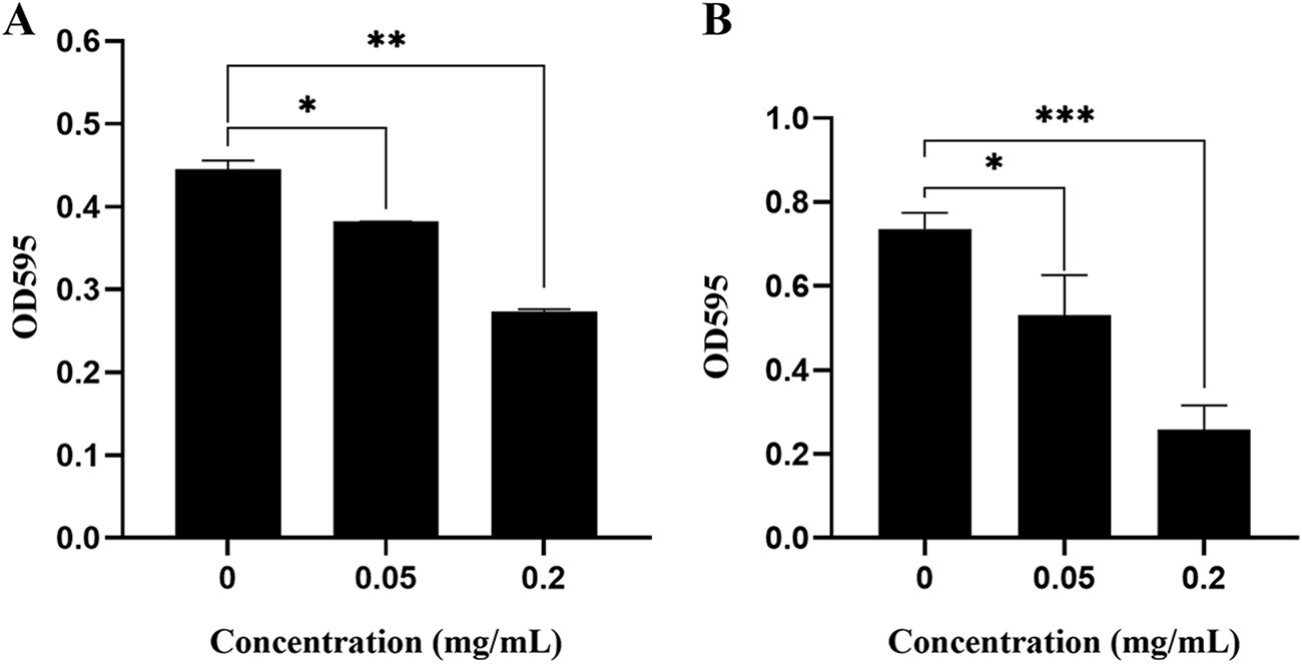
Effect of AL-3AA on P. aeruginosa PAO1 biofilm formation and eradication. (A) AL-3AA inhibited P. aeruginosa PAO1 biofilm formation. (B) AL-3AA reduces pre-formed P. aeruginosa PAO1 biofilm. Significant difference between groups is indicated by *, **, and ***, representing *P* < 0.05, *P* < 0.01, and *P* < 0.001, respectively. (N = 3).

For the biofilm formation assay, AL-3AA inhibited biofilm formation dose-dependently; 0.20 mg/mL AL-3AA could reduce biofilm formation (Fig. 2A).

To evaluate the effect of AL-3AA on eradicating or disassembling bacterial consortia, pre-formed biofilm of P. aeruginosa PAO1 was treated with AL-3AA of different concentrations. The amount of biofilm remaining (as shown by crystal violet staining) was determined at 2 h after initiation of treatment. As showed in Fig. 2B, AL-3AA decreased the amount of stained biofilm material, even 0.05 mg/mL AL-3AA could destroy the biofilm, and 0.20 mg/mL AL-3AA reduced the biofilm significantly. Many bacteria form multicellular biofilms, causing more resistance to antimicrobial reagents, and approximately 65% of all human infections are caused by biofilm-forming bacteria (26). Thus, the activity of eradicating biofilm is important for new antibiotic reagents. These results confirmed that AL-3AA acted against pre-formed biofilms and inhibited their growth.

### Structure analysis of LysPA26 and artilysins.

**FIG 3:**
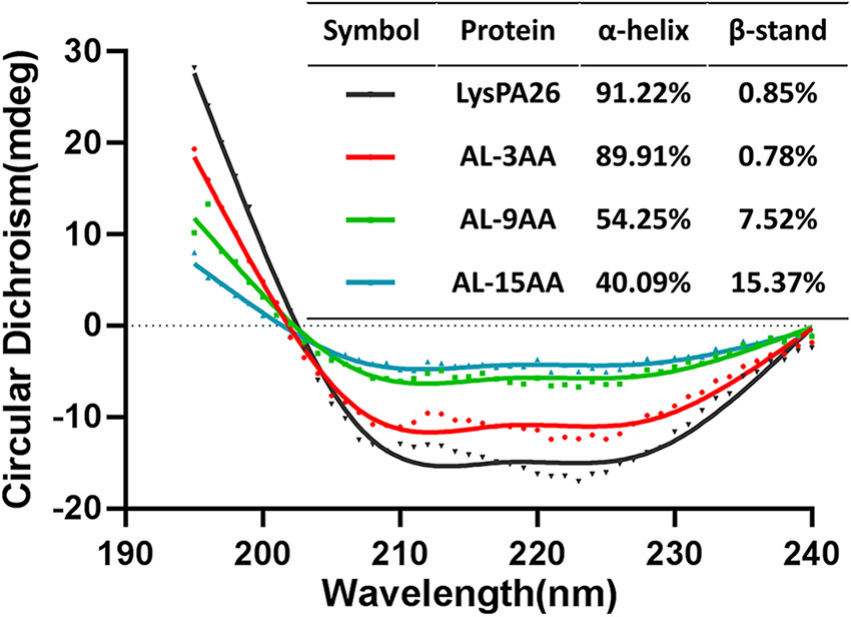
Circular dichroism (CD) spectra of AL-3AA and other proteins, indicating the conformation changes of proteins.

As circular dichroism (CD) showed (Fig. 3), LysPA26 has a mainly α-helical composition (91%) and AL-3AA does not show conformational differences compared to LysPA26. Unexpectedly, the AL-9AA and AL-15AA have low ratio of α-helical composition (54% and 40%, respectively) and increased β-sheets (7.5% and 15.37%, respectively). These results suggested the longer linker (9 or 15 amino acids) between SMAP29 and LysPA26 could impact the secondary structure of LysPA26, and thus reduce the antibacterial effect of recombinant proteins.

### AL-3AA kills P. aeruginosa through cell lysis.

**FIG 4:**
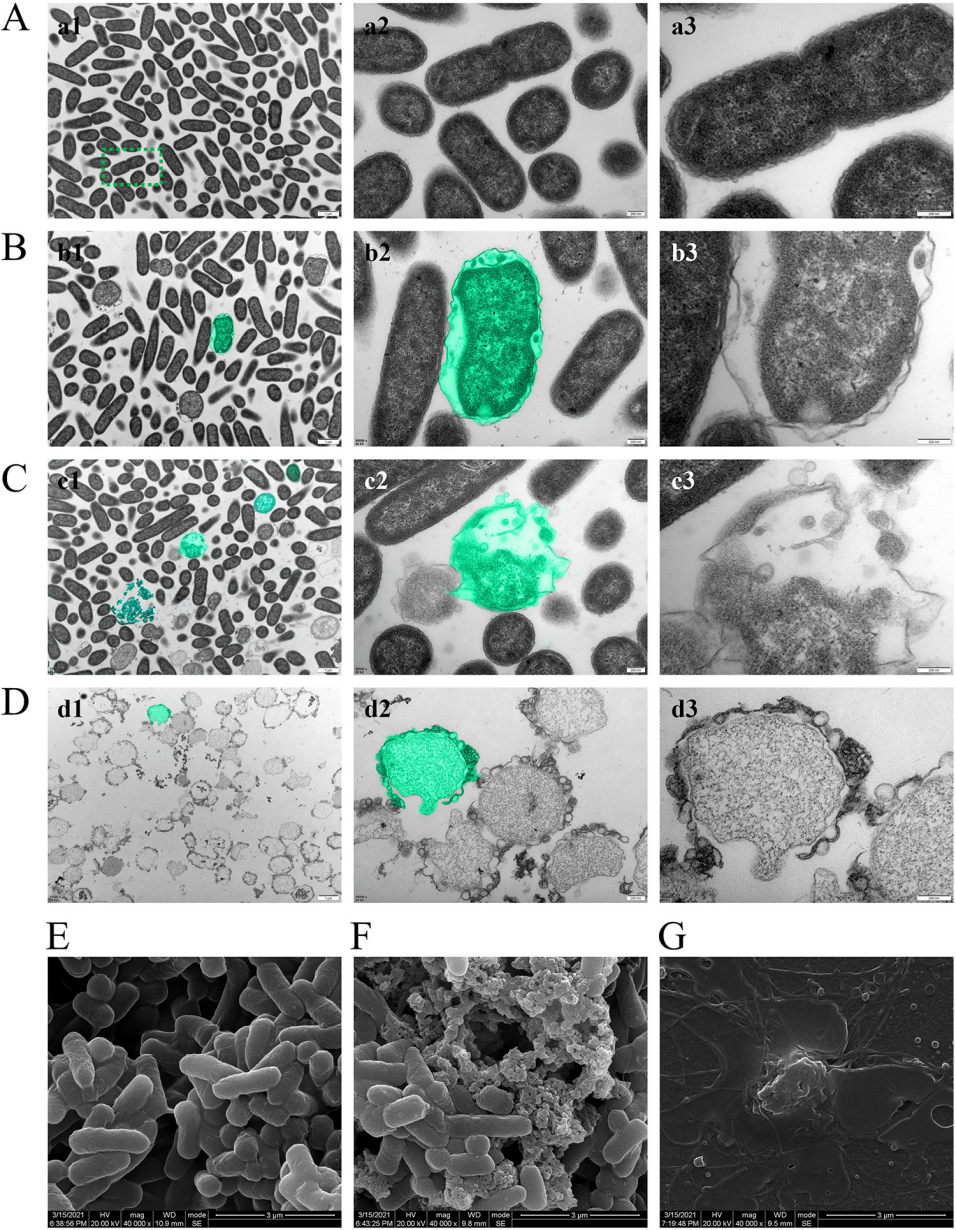
The antibacterial mechanism of AL-3AA against P. aeruginosa PAO1. TEM images of P. aeruginosa PAO1 (A to D) and SEM images of P. aeruginosa PAO1 (E to G). (A and E) Untreated P. aeruginosa PAO1. (B and F) P. aeruginosa PAO1 treated with 0.05 mg/mL LysPA26. (C) P. aeruginosa PAO1 treated with 0.50 mg/mL LysPA26. (D and G) P. aeruginosa PAO1 treated with 0.05 mg/mL AL-3AA. Scale bar in a3–d3 is 200 nm, and scale bar in E-G is 3 μm. Most of the AL-3AA treated P. aeruginosa PAO1 were lysed, the membrane was damaged, peptidoglycan layer was hydrolyzed, cytoplasmic content released, and even cells disintegrated.

The antibacterial mechanism of AL-3AA against P. aeruginosa PAO1 was investigated by morphological observation using transmission electron microscopic (TEM) and scanning electron microscope (SEM). According to TEM results, the untreated P. aeruginosa PAO1 was devoid of artifacts, bleb-like bulges, and collapsed cell structures (Fig. 4A). However, ultrastructural changes were observed in AL-3AA-treated or LysPA26-treated bacteria (Fig. 4B to 4D). The membrane of LysPA26 treated P. aeruginosa PAO1 appeared damaged, showing an undulating appearance and disorganized cell surface (Fig. 4B and C). The structures of AL-3AA treated P. aeruginosa PAO1 were damaged, and most of the cells were lysed, showing vesicles emanating from the cell wall, detachment of cellular membrane, cytoplasmic release, even cellular disintegration (Fig. 4D1 to 4D3). It was interesting that 0.05 mg/mL AL-3AA had stronger antibacterial activity (Fig. 4D) than 0.5 mg/mL LysPA26 (Fig. 4C), indicating the fusion of SMAP29 peptide at the N-terminal of LysPA26 can improve the activity significantly. Based on the SEM micrographs, significant physical damage was observed in the treated bacterial cells, such as big holes in the structure of LysPA26 treated bacterial (Fig. 4F), and complete disintegration of AL-3AA treated P. aeruginosa PAO1 (Fig. 4G). These results improved understanding of the mode of biological action of AL-3AA.

In summary, in this study a new dual target antibacterial protein was designed through the fusion of SMAP29 at the N-terminal of LysPA26 with 3 amino acids as linker, designated AL-3AA. AL-3AA has shown highly bactericidal, with more than 99.97% killing at low dose, and it kills P. aeruginosa PAO1 rapidly and dose-dependently. It also has broad-spectrum antimicrobial activity against Gram-negative bacteria, including P. aeruginosa, K. pneumoniae, and E. coli. Furthermore, AL-3AA can inhibit P. aeruginosa PAO1 biofilm formation and eradicate pre-formed biofilms. The antibacterial mechanism investigation has provided valuable information about the antibacterial action of AL-3AA, which can lyse and disintegrate the bacterial quickly. The results suggest AL-3AA could be developed as a promising candidate for anti-P. aeruginosa treatments.

## MATERIALS AND METHODS

### Strains and plasmids.

The P. aeruginosa PAO1, K. pneumoniae, E. coli, S. haemolyticus, and S. epidermidis were gifts from professor Yiwen Chu (Chengdu University). The recombinant proteins (AL-3AA, AL-9AA, and AL-15AA) were designed based on fusion of SMAP29 at N-terminal of LysPA26, with GSA, 3×GSA, and 3×GGGGS as linker, respectively. The coding sequence for AL-9AA was codon-optimized and synthesized (Qingke Zixi Biotechnology Co., Ltd., China), then inset to pET30a vector or pBV220 vector, constructing two plasmids, pET30a-His_6_-SUMO-SMAP29-(GSA)_3_-LysPA26 and pBV220-His_6_-SUMO-SMAP29-(GSA)_3_-LysPA26. The plasmids for AL-3AA or AL-15AA were constructed based on pET30a-His_6_-SUMO-SMAP29-(GSA)_3_-LysPA26 or pBV220-His_6_-SUMO-SMAP29-(GSA)_3_-LysPA26, by PCR and homologous recombination using ClonExpress II One Step Cloning Kit.

### Recombinant proteins expression and purification.

The recombinant proteins were expressed in Escherichia coli strain Transetta (DE3) cell (Transgen Biotech, China) or BL21(DE3) pLySs cell (Transgen Biotech, China). When OD_600_ reached 0.80 to 1.00, protein AL-3AA was induced at 30°C for 4 h by 0.5 mM isopropyl-β-d-thiogalactopyranoside (BBI Life Sciences, China), using pET30a-His_6_-SUMO-SMAP29-GSA-LysPA26, while the proteins AL-9AA and AL-15AA were expressed using pBV220-His_6_-SUMO-SMAP29-(GSA)_3_-LysPA26 and pBV220-His_6_-SUMO-SMAP29-(GGGGS)_3_-LysPA26, induced at 42°C for 4 h. After induction, *E.coli* cells were harvested by centrifugation at 9000 g for 6 min at 4°C. Then cells were resuspended in lysis Buffer (50 mM NaH_2_PO_4`_2H_2_O, 300 mM NaCl, pH 8.0) and lysed by a high pressure homogenizer (AH-NANO, ATS Engineering) at 800 bar. Cell lysates were centrifuged at 9000 g at 4°C for 1 h, and the supernatant was collected and filtered (0.22 μm) before purification. Firstly, the His_6_-SUMO tag fusion proteins were purified using Ni-Tanrose 6FF affinity chromatography column (Welch, China) on an AKTA pure system (GE Healthcare, USA). The proteins were bound to the column using Buffer A (50 mM NaH_2_PO_4`_2H_2_O, 300 mM NaCl, pH 8.0) and eluted with a linear gradient to 100% Buffer B (50 mM NaH_2_PO_4`_2H_2_O, 300 mM NaCl, 500 mM imidazole pH 8.0). The eluted AL-3AA were desalinated and concentrated to a volume of approximately 4 mL with Buffer A. Secondly, the His_6_-SUMO tags of purified fusion proteins were cleaved with His_6_-ULP1 enzyme, then both the His_6_-SUMO tags and His_6_-ULP1 enzyme were removed by using affinity chromatography on an AKTA pure system. Finally, AL-3AA, AL-9AA, and AL-15AA proteins were concentrated and the protein concentration was determined by modified BCA Protein assay kit (Sangon Biotech, China). The purification and integrity of the recombinant proteins were checked by SDS-PAGE. All samples were stored at −80°C until use.

### Circular dichroism (CD) analysis.

CD spectra were determined by the spectro-polarmeter (Chirascan Plus, Applied Photophysics, United Kingdom). Protein was diluted to 0.1 mg/mL with ultra-pure water. The sample was assayed from 195 nm to 260 nm in 1 mm path length cell at 23°C. The spectra were recorded and each sample was measured three times. To determine the proportion of secondary structure, the data were analyzed by K2D3 online analysis program (27).

### Antibacterial assay.

To evaluate the antimicrobial activity of these proteins, a microdilution method was performed. Briefly, P. aeruginosa PAO1 was grown in LB broth at 37°C for 3h. Bacterial cells were harvested by centrifugation (12000 rpm for 3 min) and washed once with 1×PBS (pH 7.4). The cell pellet was resuspended with dilution buffer (10 mM HEPES with or without EDTA, pH 7.4) and adjusted OD_600_ to 1.0 ± 0.05 to a final density of 2 × 10^8^ ∼ 8 × 10^8^ CFU/mL. These proteins were diluted to 0.10 mg/mL, and added into an equal volume of P. aeruginosa PAO1, making the final concentration of these proteins at 0.05 mg/mL. After incubation at 37°C for 60 min, the mixture was serially diluted and plated on LB agar in triplicate and cultured at 37°C. After incubation, P. aeruginosa PAO1 colonies were counted. The antibacterial activity was quantified as the relative inactivation in logarithmic units, lg(N_0_/N_i_), where N_0_ is the initial number of untreated cells (in the negative control) and N_i_ is the number of residual cells counted after treatment with different proteins (28). The antimicrobial activity of AL-3AA against K. pneumoniae, E. coli, S. haemolyticus, and S. epidermidis was determined similarly, and the concentration gradient of AL-3AA was increased to 0.05 mg/mL and 0.10 mg/mL without EDTA.

### Concentration-kill curve and time-kill curve assay.

For the concentration-kill curve assay, P. aeruginosa PAO1 was incubated with AL-3AA of gradient concentrations from 0.01 mg/mL to 0.10 mg/mL at 37°C for 60 min. The antibacterial activity was measured as mentioned above.

For the time-kill curve assay, P. aeruginosa PAO1 was incubated with 0.05 mg/mL AL-3AA or 0.05 mg/mL LysPA26 at 37°C for different times, from 2 min to 30 min. At predetermined time points, 100 μl of the incubated P. aeruginosa PAO1 was taken, serially diluted, and plated on LB agar in triplicate. The antibacterial activity was measured as mentioned above.

### Measurement of biofilm formation and eradication *in vitro*.

To evaluate the effect of AL-3AA on growth of P. aeruginosa PAO1 biofilms, a serial dilution of AL-3AA was prepared. Strains were cultured aerobically overnight in LB and diluted to an OD_600_ of 0.2 into fresh LB broth with 2% glucose. These bacterial suspensions were dispensed into the 1.5 ml EP tubes and every three tubes as biological replicates were treated by AL-3AA with final concentration at 0.05 mg/mL or 0.20 mg/mL, then incubated statically at 37°C under aerobic conditions. Following 2 days culture, the medium was discarded, and the EP tubes were gently washed three times with water and methanol-fixed for 15 min, and the bacterial in tubes were stained with 500 μL of 1% (wt/vol) crystal violet in ethanol for 10 min. Tubes were rinsed three times with water, then adhered crystal violet-stained material (biofilm) was dissolved in 500 μL destaining solution (33% [vol/vol] acetic acid) and quantified by reading absorbance at 595 nm (29). For the cellular content analysis of pre-formed biofilms after treatment with AL-3AA solutions, biofilms in LB were grown for 2 days. The medium was then removed and replaced with fresh PBS or AL-3AA dilution with PBS. EP tubes were then incubated aerobically at 37°C and stained with crystal violet as described above at 2 h post-treatment (29).

### The bactericidal mechanism.

To illustrate the bactericidal mechanism of AL-3AA, TEM and SEM analysis were performed. P. aeruginosa PAO1 was cultured in LB broth and adjusted OD600 to 1.0 ± 0.05.

For TEM analysis, P. aeruginosa PAO1 was incubated with 0.05 mg/mL AL-3AA, 0.05 mg/mL LysPA26, 0.5 mg/mL LysPA26, or buffer without protein at 37°C for 20 min. After incubation, the protein-treated or untreated (negative control) P. aeruginosa PAO1 was placed on a carbon-coated copper grid. The samples were stained with uranyl acetate for 10∼15 min, followed by lead citrate staining for 1∼2 min, and observed by transmission electron microscopy (TEM, JEM-1400FLASH, JEOL, Japan) at 80 kV.

For SEM analysis, P. aeruginosa PAO1 was incubated with 0.05 mg/mL AL-3AA, 0.05 mg/mL LysPA26, or buffer without protein at 37°C for 20 min. After incubation, the protein-treated or untreated (negative control) P. aeruginosa PAO1 was sputter coated (Sputter Coater, E-1045, Hitachi, Japan) and introduced into the vacuum chamber of a scanning electron microscope (SEM, Inspect, FEI NanoPorts, American). A series of micro-photographs were taken at a magnification of ×10000, ×20000, and ×40000 for viewing the surface morphology.

### Statistical analysis.

Data are presented as the mean ± standard error, and *n* = 3 for each group; data were analyzed by GraphPad Prism 8. Statistical analyses were performed by T-tests (equal variance) for two different groups. Significant difference between groups was indicated by *, **, ***, and ****, representing *P* < 0.05, *P* < 0.01, *P* < 0.001, and *P* < 0.0001, respectively.

## References

[1] Burnham CD, Leeds J, Nordmann P, O'Grady J, Patel J. 2017. Diagnosing antimicrobial resistance. Nat Rev Microbiol 15:697–703. doi:10.1038/nrmicro.2017.103. 29021600

[2] Sugden R, Kelly R, Davies S. 2016. Combatting antimicrobial resistance globally. Nat Microbiol 1:16187. doi:10.1038/nmicrobiol.2016.187. 27670123

[3] Laxminarayan R, Sridhar D, Blaser M, Wang M, Woolhouse M. 2016. Achieving global targets for antimicrobial resistance. Science 353:874–875. doi:10.1126/science.aaf9286. 27540009

[4] Doi Y. 2019. Treatment options for carbapenem-resistant Gram-negative bacterial infections. Clin Infect Dis 69:S565–S575. doi:10.1093/cid/ciz830. 31724043 PMC6853760

[5] Makharita RR, El-Kholy I, Hetta HF, Abdelaziz MH, Hagagy FI, Ahmed AA, Algammal AM. 2020. Antibiogram and genetic characterization of carbapenem-resistant Gram-negative pathogens incriminated in healthcare-associated infections. Infect Drug Resist 13:3991–4002. doi:10.2147/IDR.S276975. 33177849 PMC7649219

[6] Theuretzbacher U, Gottwalt S, Beyer P, Butler M, Czaplewski L, Lienhardt C, Moja L, Paul M, Paulin S, Rex JH, Silver LL, Spigelman M, Thwaites GE, Paccaud JP, Harbarth S. 2019. Analysis of the clinical antibacterial and antituberculosis pipeline. Lancet Infect Dis 19:e40–e50. doi:10.1016/S1473-3099(18)30513-9. 30337260

[7] Theuretzbacher U, Outterson K, Engel A, Karlen A. 2020. The global preclinical antibacterial pipeline. Nat Rev Microbiol 18:275–285. doi:10.1038/s41579-019-0288-0. 31745331 PMC7223541

[8] Fischbach MA, Walsh CT. 2009. Antibiotics for emerging pathogens. Science 325:1089–1093. doi:10.1126/science.1176667. 19713519 PMC2802854

[9] Heselpoth R, Euler C, Schuch R, Fischetti V. 2019. Lysocins: bioengineered antimicrobials that deliver lysins across the outer membrane of Gram-negative bacteria. Antimicrob Agents Chemother 63:e00342-19. 30962344 10.1128/AAC.00342-19 PMC6535517

[10] McCallin S, Sacher JC, Zheng J, Chan BK. 2019. Current state of compassionate phage therapy. Viruses 11:343. doi:10.3390/v11040343. 31013833 PMC6521059

[11] Cafora M, Deflorian G, Forti F, Ferrari L, Binelli G, Briani F, Ghisotti D, Pistocchi A. 2019. Phage therapy against *Pseudomonas aeruginosa* infections in a cystic fibrosis zebrafish model. Sci Rep 9:1527. doi:10.1038/s41598-018-37636-x. 30728389 PMC6365511

[12] Łusiak-Szelachowska M, Zaczek M, Weber-Dąbrowska B, Międzybrodzki R, Kłak M, Fortuna W, Letkiewicz S, Rogóż P, Szufnarowski K, Jończyk-Matysiak E, Owczarek B, Górski A. 2014. Phage neutralization by sera of patients receiving phage therapy. Viral Immunol 27:295–304. doi:10.1089/vim.2013.0128. 24893003 PMC4076984

[13] Abdelkader K, Gerstmans H, Saafan A, Dishisha T, Briers Y. 2019. The preclinical and clinical progress of bacteriophages and their lytic enzymes: the parts are easier than the whole. Viruses 11:96. doi:10.3390/v11020096. 30678377 PMC6409994

[14] Oliveira H, Vilas Boas D, Mesnage S, Kluskens LD, Lavigne R, Sillankorva S, Secundo F, Azeredo J. 2016. Structural and enzymatic characterization of ABgp46, a novel phage endolysin with broad anti-Gram-negative bacterial activity. Front Microbiol 7:208. doi:10.3389/fmicb.2016.00208. 26955368 PMC4768612

[15] Kashani HH, Schmelcher M, Sabzalipoor H, Hosseini ES, Moniri R. 2018. Recombinant endolysins as potential therapeutics against antibiotic-resistant *Staphylococcus aureus*: current status of research and novel delivery strategies. Clin Microbiol Rev 31:e00071-17. doi:10.1128/CMR.00071-17. 29187396 PMC5740972

[16] Guo M, Feng C, Ren J, Zhuang X, Zhang Y, Zhu Y, Dong K, He P, Guo X, Qin J. 2017. A novel antimicrobial endolysin, LysPA26, against *Pseudomonas aeruginosa*. Front Microbiol 8:293. doi:10.3389/fmicb.2017.00293. 28289407 PMC5326749

[17] Sao-Jose C. 2018. Engineering of phage-derived lytic enzymes: improving their potential as antimicrobials. Antibiotics (Basel) 7:29. doi:10.3390/antibiotics7020029. 29565804 PMC6023083

[18] Catalao MJ, Gil F, Moniz-Pereira J, Sao-Jose C, Pimentel M. 2013. Diversity in bacterial lysis systems: bacteriophages show the way. FEMS Microbiol Rev 37:554–571. doi:10.1111/1574-6976.12006. 23043507

[19] Young R. 2014. Phage lysis: three steps, three choices, one outcome. J Microbiol 52:243–258. doi:10.1007/s12275-014-4087-z. 24585055 PMC4012431

[20] Paradis-Bleau C, Cloutier I, Lemieux L, Sanschagrin F, Laroche J, Auger M, Garnier A, Levesque RC. 2007. Peptidoglycan lytic activity of the *Pseudomonas aeruginosa* phage phiKZ gp144 lytic transglycosylase. FEMS Microbiol Lett 266:201–209. doi:10.1111/j.1574-6968.2006.00523.x. 17233731

[21] Lee MT, Chen FY, Huang HW. 2004. Energetics of pore formation induced by membrane active peptides. Biochemistry 43:3590–3599. doi:10.1021/bi036153r. 15035629

[22] Matsuzaki K, Murase O, Fujii N, Miyajima K. 1996. An antimicrobial peptide, magainin 2, induced rapid flip-flop of phospholipids coupled with pore formation and peptide translocation. Biochemistry 35:11361–11368. doi:10.1021/bi960016v. 8784191

[23] Oren Z, Shai Y. 1998. Mode of action of linear amphipathic α-helical antimicrobial peptides. Biopolymers 47:451–463. doi:10.1002/(SICI)1097-0282(1998)47:6<451::AID-BIP4>3.0.CO;2-F. 10333737

[24] Yan J, Wang K, Dang W, Chen R, Xie J, Zhang B, Song J, Wang R. 2013. Two hits are better than one: membrane-active and DNA binding-related double-action mechanism of NK-18, a novel antimicrobial peptide derived from mammalian NK-lysin. Antimicrob Agents Chemother 57:220–228. doi:10.1128/AAC.01619-12. 23089755 PMC3535945

[25] Briers Y, Walmagh M, Grymonprez B, Biebl M, Pirnay JP, Defraine V, Michiels J, Cenens W, Aertsen A, Miller S, Lavigne R. 2014. Art-175 is a highly efficient antibacterial against multidrug-resistant strains and persisters of *Pseudomonas aeruginosa*. Antimicrob Agents Chemother 58:3774–3784. doi:10.1128/AAC.02668-14. 24752267 PMC4068523

[26] Stewart PS, Costerton JW. 2001. Antibiotic resistance of bacteria in biofilms. Lancet 358:135–138. doi:10.1016/s0140-6736(01)05321-1. 11463434

[27] Louis-Jeune C, Andrade-Navarro MA, Perez-Iratxeta C. 2012. Prediction of protein secondary structure from circular dichroism using theoretically derived spectra. Proteins Structure Function & Bioinformatics 80:374–381. doi:10.1002/prot.23188. 22095872

[28] Plotka M, Kapusta M, Dorawa S, Kaczorowska AK, Kaczorowski T. 2019. Ts2631 Endolysin from the extremophilic *Thermus scotoductus* bacteriophage vB_Tsc2631 as an antimicrobial agent against Gram-negative multidrug-resistant bacteria. Viruses 11:657. doi:10.3390/v11070657. 31323845 PMC6669862

[29] Behroozian S, Svensson S, Li L, Davies J. 2020. Broad-spectrum antimicrobial and antibiofilm activity of a natural clay mineral from British Columbia, Canada. mBio 11:e02350-20. 33024043 10.1128/mBio.02350-20 PMC7542368

