# Supplementary material for: Design SMAP29-LysPA26 as a Highly Efficient Artilysin against Pseudomonas aeruginosa with Bactericidal and Antibiofilm Activity
Source: Microbiol Spectr. 2021 Dec 8;9(3):e00546-21. doi: 10.1128/Spectrum.00546-21 (PMC8653812; doi:10.1128/Spectrum.00546-21)
Supplement: SUPPLEMENTAL FILE 1 — Supplemental material. Download SPECTRUM00546-21_Supp_1_seq8.pdf, PDF file, 0.3 MB [file spectrum00546-21_supp_1_seq8.pdf]

Supplemental Material

Design SMAP29-LysPA26 as a Highly efficient artilysin against *Pseudomonas aeruginosa* with Bactericidal and Antibiofilm Activity

Tingting Wang[a,b,1], Yongxiang Zheng[a,b,1], Jiami Dai[a,b], Junxiu Zhou[a,b], Rong Yu[a,b], Chun Zhang[a,b*]

[a]Department of Biopharmaceutics, West China School of Pharmacy, Sichuan University, Chengdu 610041, PR China

[b]Key Laboratory of Drug-Targeting and Drug Delivery System of the Education Ministry, Sichuan Engineering Laboratory for Plant-Sourced Drug and Sichuan Research Center for Drug Precision Industrial Technology, West China School of Pharmacy Sichuan University, Chengdu, 610041 PR China

[1] Tingting Wang and Yongxiang Zheng contributed equally to this work. Author order was determined both alphabetically and in order of increasing seniority.

[*]Corresponding author E-mail addresses: chunzhang@scu.edu.cn.

[*]Corresponding author at: Department of Biopharmaceutics, West China School of Pharmacy, Sichuan University, NO.17, Renmin Road, Chengdu, Sichuan Province, 610041, P.R. China.

Content

Figure S1. The relative antibiotic activity of AL-3AA, AL-9AA and AL-15AA compared with LysPA26.

Figure S2. The impact of EDTA on the antibiotic activity of LysPA26 and AL-3AA.

Figure S3. The relative antibiotic activity of AL-3AA compared with LysPA26 at different incubation time.

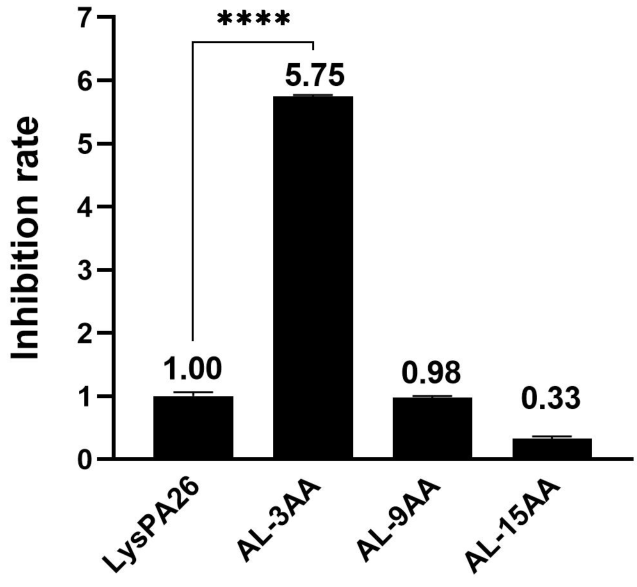

Figure S1. The relative antibiotic activity of AL-3AA, AL-9AA and AL-15AA compared with LysPA26. (N=3, Significant difference between groups was indicated by *, **, *** and ****, representing $p < 0.05$, $p < 0.01$, $p < 0.001$ and $p < 0.0001$, respectively.)

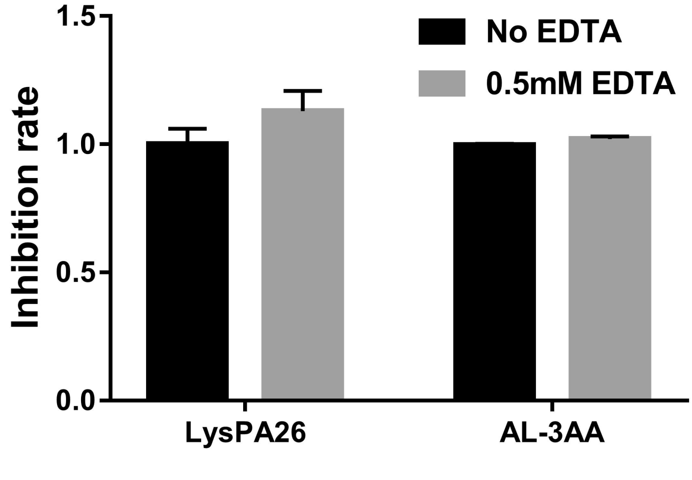

Figure S2. The impact of EDTA on the antibiotic activity of LysPA26 and AL-3AA.

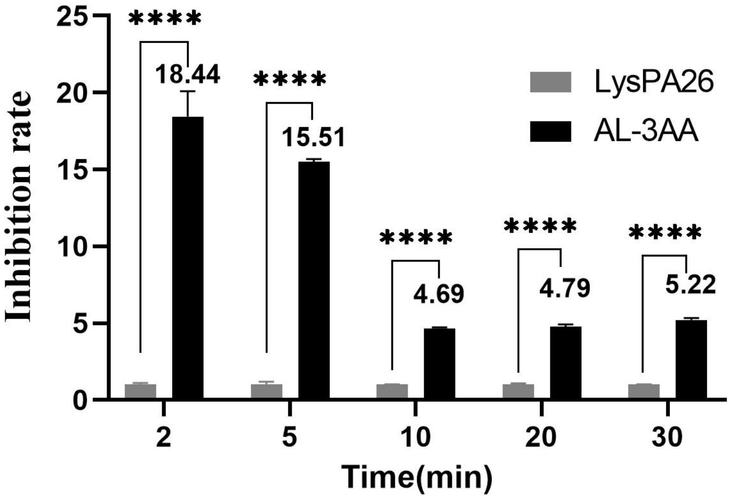

Figure S3. The relative antibiotic activity of AL-3AA compared with LysPA26 at different incubation time. (N=3, Significant difference between groups was indicated by *, **, *** and ****, representing $p < 0.05$, $p < 0.01$, $p < 0.001$ and $p < 0.0001$, respectively.)
